# Cytotoxic Effects of Doxorubicin on Cancer Cells and Macrophages Depend Differently on the Microcarrier Structure

**DOI:** 10.3390/pharmaceutics16060785

**Published:** 2024-06-09

**Authors:** Daria Kalenichenko, Irina Kriukova, Alexander Karaulov, Igor Nabiev, Alyona Sukhanova

**Affiliations:** 1 Université de Reims Champagne-Ardenne, BIOSPECT, 51100 Reims, France; daria.kalenichenko@univ-reims.fr; 2 Life Improvement by Future Technologies (LIFT) Center, Skolkovo, 143025 Moscow, Russia; irina.kryukova.mephi@gmail.com; 3 Laboratory of Nano-Bioengineering, National Research Nuclear University MEPhI (Moscow Engineering Physics Institute), 115409 Moscow, Russia; 4 Department of Clinical Immunology and Allergology, Institute of Molecular Medicine, Sechenov First Moscow State Medical University (Sechenov University), 119146 Moscow, Russia; drkaraulov@mail.ru

**Keywords:** microparticles, microcapsules, doxorubicin, cancer cells, macrophages

## Abstract

Microparticles are versatile carriers for controlled drug delivery in personalized, targeted therapy of various diseases, including cancer. The tumor microenvironment contains different infiltrating cells, including immune cells, which can affect the efficacy of antitumor drugs. Here, prototype microparticle-based systems for the delivery of the antitumor drug doxorubicin (DOX) were developed, and their cytotoxic effects on human epidermoid carcinoma cells and macrophages derived from human leukemia monocytic cells were compared in vitro. DOX-containing calcium carbonate microparticles with or without a protective polyelectrolyte shell and polyelectrolyte microcapsules of about 2.4–2.5 μm in size were obtained through coprecipitation and spontaneous loading. All the microstructures exhibited a prolonged release of DOX. An estimation of the cytotoxicity of the DOX-containing microstructures showed that the encapsulation of DOX decreased its toxicity to macrophages and delayed the cytotoxic effect against tumor cells. The DOX-containing calcium carbonate microparticles with a protective polyelectrolyte shell were more toxic to the cancer cells than DOX-containing polyelectrolyte microcapsules, whereas, for the macrophages, the microcapsules were most toxic. It is concluded that DOX-containing core/shell microparticles with an eight-layer polyelectrolyte shell are optimal drug microcarriers due to their low toxicity to immune cells, even upon prolonged incubation, and strong delayed cytotoxicity against tumor cells.

## 1. Introduction

Cancer is the leading cause of death worldwide, accounting for almost 10 million deaths in 2020, or about one in six deaths [1]. A malignant tumor is a complex “ecosystem” consisting of cancer cells, as well as infiltrating immune, endothelial, and stromal cells. There is increasing evidence that the tumor microenvironment is involved in many oncogenic processes, including tumor cell proliferation and survival, immune evasion, metastatic process, angiogenesis, and resistance to therapy. Thus, the tumor microenvironment plays a key role in tumor development and drug resistance [2,3,4,5]. Therefore, chemotherapy, one of the most effective treatments, has a number of inherent drawbacks and limitations, with low selectivity of the drugs toward cancer cells being the most critical of them [6,7]. The development of controlled and targeted antitumor-drug delivery systems is one of the challenges of personalized cancer therapy. Controlled delivery and release could reduce the side effects of antitumor drugs and their toxicity to normal cells while ensuring selectivity for cancer cells [8,9,10].

Multilayer polymer microstructures have been shown to be promising candidate carriers for targeted delivery and the modified release of drugs, as well as contrast and fluorescent detection probes for the in vitro and in vivo imaging of the delivery system [11,12,13,14,15]. Currently, this is one of the most promising approaches in the field of personalized tumor diagnosis and therapy.

Doxorubicin (DOX) is a common antitumor antibiotic of the anthracycline group, widely used in the chemotherapy of various primary and metastatic cancers [16]. Specifically, DOX can be used for chemotherapy of most types of invasive breast cancer, including triple-negative breast cancer. It can also be used together with targeted drugs, such as trastuzumab (Herceptin^®^), in the treatment of HER2-positive breast cancer. Despite its proven high efficacy in the treatment of cancer, DOX has a wide range of undesirable side effects, including strong cardiotoxicity [17,18]. Due to its high amphiphilicity and its fluorescent properties, DOX may be a useful model anticancer drug for incorporation into microcarriers in order to obtain an effective delivery system. Encapsulation of DOX in microcarriers, together with targeted delivery to the tumor site, can ensure a controlled release of the drug, thereby reducing its side effects on normal cells [19,20]. We have previously discussed the difficulty of DOX encapsulation using traditional methods (the emulsion method and the addition of organic solvents) [21,22]. The novelty of our approach to the preparation of DOX-containing microstructures is the efficient encapsulation of DOX in the aqueous phase without the use of additional components or equipment.

Optimal selection of the physicochemical properties of microstructures, such as their shape, size, and structure (the number of polymer layers in the shell, the presence or absence of a core, integration of other functional components, etc.) [23], can contribute to a prolonged release of the antitumor agent [19,24,25], an increased time of its circulation in the body, and decreased side effects on healthy tissues and organs [26], as well as ensure its targeted delivery to the tumor site without loss of its pharmacological properties [27].

The mechanical properties of the particles, including their stiffness and surface characteristics, may also influence their behavior and interaction with cells [28,29]. The rigidity of the microstructures significantly affects their internalization by cancer cells: rigid or strengthened particles are uptaken more rapidly than soft ones [28]. It has been found that the cellular uptake and subsequent endosomal transport of biodegradable and non-biodegradable microstructures strongly depend on the particle stiffness rather than the shell composition. At the same time, the rate of release of encapsulated components from microstructures may be influenced by the composition of the polymer shell. The shell of the microstructures containing non-degradable polymers, such as poly(sodium 4-styrene sulfonate) (PSS) and poly(allylamine hydrochloride) (PAH), exhibited a pH sensitivity in a pH range from 3.0 to 7.5. The experiments have shown that the shell of these microstructures is highly permeable in the slightly acidic tumor microenvironment (6.5–6.8) [15,30].

Therefore, in this study, we prepared different types of microstructures—calcium carbonate microbeads (MBs) (rigid microstructures), MBs coated with layers of oppositely charged polyelectrolytes (PAH and PSS) (rigid microstructures with a polymer shell), and polyelectrolyte microcapsules (MCs) (soft microstructures) containing DOX—in order to determine how the structure of the microcarriers affect their cytotoxicity against human tumor cells and immune cells (macrophages) when in vitro.

## 2. Materials and Methods

### 2.1. Materials

Sodium chloride (NaCl), sodium carbonate (Na_2_CO_3_), calcium chloride (CaCl_2_), glycerol, poly(allylamine hydrochloride) (PAH, Mw ≈ 17,500 Da), poly(sodium-4-styrenesulfonate) (PSS, Mw ≈ 70,000 Da), phorbol 12-myristate 13-acetate (PMA), dimethylsulfoxide (DMSO), and DOX (suitable for fluorescence, 98.0–102.0%) were purchased from Sigma-Aldrich Chimie S.a.r.l. (Merck), Saint-Quentin-Fallavier, France. UltraPure™ 0.5 M EDTA (pH 8.0) was purchased from Thermo Fischer Scientific, Illkirch, France.

All polymer and buffer solutions were prepared using Milli-Q water (18.2 mΩ·cm) and additionally filtered through the sterile Millex-GV filters (0.22 μm) obtained from Sigma-Aldrich Chimie S.a.r.l. (Merck), Saint-Quentin-Fallavier, France.

Roswell Park Memorial Institute (RPMI) 1640 medium with phenol red and without L-glutamine, heat-inactivated fetal bovine serum (FBS), 10,000 U/mL of solution of penicillin–streptomycin, 100 mM solution of sodium pyruvate, 200 mM solution of L-glutamine, Dulbecco’s phosphate-buffered saline (DPBS), sterile PBS (pH 7.4), (3-(4,5-dimethylthiazol-2-yl)-2,5-diphenyltetrazolium bromide) (MTT), and 0.05% solution of Trypsin-EDTA were purchased from Thermo Fischer Scientific, Illkirch, France. 

The A-431 human epidermoid carcinoma cell line was obtained from ATCC. The THP-1 human leukemia monocytic cell line was kindly provided by Prof. Halima Kerdjoudj (EA-4691 BIOS, Université de Reims Champagne-Ardenne, Reims, France).

### 2.2. Methods

#### 2.2.1. Fabrication of Microstructures of Different Types

##### Synthesis of Calcium Carbonate Microbeads

Calcium carbonate MBs were further used as cores for the assembly of core/shell microstructures, and the microcapsules were obtained by mixing 7.5 mL of 0.33 M Na_2_CO_3_ and 7.5 mL of 0.33 M CaCl_2_ with an equivalent volume of 44 wt% aqueous solution of glycerol (Sigma-Aldrich Chimie S.a.r.l. (Merck), Saint-Quentin-Fallavier, France) serving as a thickening agent, as described earlier [22]. The reaction mixture was placed onto a magnetic stirrer at 500 rpm for 60 min. The obtained MBs were washed to remove excess glycerol four times with ultrapure water by sequential centrifugation at 3000× *g* for 15 min. After the final centrifugation, the resultant MB precipitate was dried at 90 °C overnight.

##### Preparation of Core/Shell Microparticles and Microcapsules

Core/shell microparticles consisting of the MBs coated with eight-layer polymer shells (MB(+8L)) and MC consisting of the polymer shell alone (MC(8L)) were obtained by means of layer-by-layer adsorption of oppositely charged polyelectrolytes (the polycation PAH and the polyanion PSS) onto the MB surface [22,23,31].

About 10^8^ MBs, dried after the synthesis, were resuspended in 0.5 mL of ultrapure water. The suspension was sonicated on an ultrasonic bath to separate the aggregated particles. Then, 0.5 mL of a PAH solution (2 mg/mL) in 0.5 M NaCl was added to 0.5 mL of the suspension. The resulting mixture was stirred on a vortex and sonicated on an ultrasonic bath for 60 s. The suspension was incubated on a rotary shaker for 20 min at room temperature and then centrifuged at 1377× *g* for 3 min. The supernatant was withdrawn, and the pellet was resuspended in 0.5 mL of water. To apply the next layer, 0.5 mL of a PSS solution (2 mg/mL) in 0.5 M NaCl was added to 0.5 mL of the mixture. The suspension was sonicated and incubated under the conditions described above. The microstructures were washed to remove excess polyelectrolyte three times with ultrapure water by centrifugation at 1377× *g* for 3 min. The polyelectrolytes were applied onto the MB surface in the following order: PAH/PSS/PAH/PSS/PAH/PSS/PAH/PSS.

After the last layer was applied and the last washing step was performed, the obtained MBs(+8L) were resuspended in 0.5 mL of ultrapure water and stored at +4 °C until use.

The hollow MC(8L) was obtained by incubating 10^7^ MB(+8L) in 0.5 M EDTA (pH 8.0) (Thermo Fischer Scientific, Illkirch, France) for 4 h to remove the calcium carbonate core. The resulting MC(8L) was sedimented by centrifugation for 5 min at 8609× *g* and resuspended in ultrapure water. The washing with ultrapure water was repeated three more times; after the last washing, the MC(8L) was resuspended in 0.5 mL of water.

The size distributions of the prepared microstructures were analyzed by means of dynamic light scattering using a Zetasizer NanoZS (Malvern Panalytical, Palaiseau, France). The deposition of polyelectrolytes was controlled by means of laser Doppler electrophoresis using a Zetasizer NanoZS. Each measurement was made at least five times, and the results were estimated using standard statistical methods.

##### Loading of Doxorubicin into the Microstructures

The DOX-containing MBs were obtained by coprecipitation at the step of MB synthesis. First, 1 mL of a 10 mg/mL DOX solution was added to 14.5 mL of a mixture of 0.33 M CaCl_2_ and 44 wt% glycerol. The resulting mixture was stirred on a magnetic stirrer, and then 14.5 mL of a mixture of 0.33 M Na_2_CO_3_ and 44 wt% glycerol was added. The reaction mixture was stirred for 60 min at 500 rpm. The synthesized MB-DOX were washed from the residual reaction mixture two times with ultrapure water. The obtained MB-DOX precipitate was dried at 90 °C overnight.

The MB-DOX were subsequently used as substrates to obtain core/shell microparticles containing DOX (MB(+8L)-DOX). They were also obtained through layer-by-layer adsorption of polyelectrolytes, as described above. The polyelectrolytes were applied in the order PAH/PSS/PAH/PSS/PAH/PSS/PAH/PSS.

The DOX-containing microcapsules (MC(8L)-DOX) were obtained via spontaneous loading of the anticancer drug into the MC(8L). For this purpose, 0.5 mL of a mixture of 0.05 M phosphate buffer solution (pH 8.0) containing 0.5 M NaCl and 0.032 mg/mL DOX was added to a precipitate containing ~6 × 10^6^ previously obtained MC(8L). The suspension was incubated for 16 h at 25 °C on a rotary shaker in test tubes wrapped in foil. After incubation, the sample was centrifuged at 8609× *g* for 5 min, the supernatant was withdrawn, and the resulting MC(8L)-DOX was resuspended in 0.5 mL of ultrapure water.

The amount of DOX loaded into MB, MB(+8L), and MC(8L) was determined spectrophotometrically at the wavelength of the maximum absorption of DOX (485 nm) using a Spark^TM^ 10M model of multimode microplate reader Tecan (Männedorf, Switzerland) as described earlier [22].

The release of DOX from the obtained microstructures was analyzed under the following physiological conditions: a temperature of 37 °C and a pH of 7.4. For this purpose, samples containing 6 × 10^6^ microstructures in the release medium (0.05 M phosphate buffer solution, pH 7.4) were incubated at 37 °C under constant stirring on a shaker at 500 rpm, the supernatants were collected at fixed time intervals (45 min, 1.5 h, 3 h, 6 h, 12 h, 24 h, 48 h, and 72 h) by centrifugation at 1900× *g* for 10 min, and the DOX content of the samples was determined by spectrophotometry at the wavelength of the maximum absorbance of DOX (485 nm).

The size distributions of the DOX-containing microstructures were analyzed by dynamic light scattering using a Zetasizer NanoZS (Malvern Panalytical, Palaiseau, France). The deposition of polyelectrolytes was monitored by laser Doppler electrophoresis using a Zetasizer NanoZS. Each measurement was made at least five times, and the results were estimated using standard statistical methods.

#### 2.2.2. Scanning Electron Microscopy

A scanning electron microscope with an SU8030 field emission gun (Hitachi, Tokyo, Japan) at the NANO’MAT platform (University of Technology of Troyes, Troyes, France) was used. The powder of dried microstructures was applied onto a conductive carbon adhesive tape and scanned at an accelerating voltage of 3.0 kV, a working distance of 8.5–8.6 mm, and an emission current of 9000 nA.

#### 2.2.3. Cell Culture

Human epidermoid carcinoma A-431 cells were cultured in a complete RPMI-1640 medium supplemented with 10% heat-inactivated FBS, 2 mM L-glutamine, 1% penicillin–streptomycin solution, and 0.1% sodium pyruvate at 37 °C (Thermo Fischer Scientific, Illkirch, France) in a 5% CO_2_ atmosphere under sterile conditions. THP-1 macrophages were obtained by incubating THP-1 human monocytic leukemia cells in a complete RPMI-1640 medium supplemented with 150 ng/mL of PMA for 48 h at 37 °C in a 5% CO_2_ atmosphere. After PMA stimulation, the THP-1 cells were cultured in a complete RPMI-1640 medium. When the cells had formed a monolayer, they were detached from culture flasks with a 0.05% Trypsin–EDTA solution (Thermo Fischer Scientific, Illkirch, France). The cell suspension was centrifuged at 302× *g* for 5 min, the cell pellet was resuspended in complete growth medium, and the cells were counted in a KOVA™Glasstic™ slide (Thermo Fisher Scientific, Illkirch, France) and placed into a fresh culture flask. Both cell lines were cultured for no more than 20 passages.

#### 2.2.4. MTT Assay

Cell viability was estimated using the MTT assay according to the manufacturer’s instructions (Thermo Fischer Scientific, Illkirch, France). The cells were seeded into a 96-well microplate, ~3.2 × 10^4^ cells per well (in 0.18 mL of complete working medium) in the case of A-431 cells and ~5.3 × 10^4^ cells per well in the case of differentiated THP-1m cells. These amounts were so selected that confluence would be achieved within 24 h of incubation. The cells were incubated under sterile conditions at 37 °C in an atmosphere of 5% CO_2_.

After 80% confluence was reached, 0.2 mL of the sample suspension in the complete medium was added to the microplate wells. The samples tested are listed below.

- Microstructures containing DOX in the final concentration range from 0 to 9 µM: MB-DOX; MB(+8L)-DOX; MC(8L)-DOX.
- Microstructures not containing DOX (control samples) at a ratio from 0 to 50 microstructures per cell: MB; MB(+8L); MC(8L).
- A DOX solution in the concentration range from 0 to 9 µM in the complete medium.

Wells containing only 0.2 mL of the complete working medium and empty (blank) wells were also used as controls. Each experiment was repeated three times in three replicates.

After incubation for 24 h, 48 h, 72 h, or 96 h, 0.02 mL of a 12 mM MTT solution was added to the microplate wells, and the microplates were incubated for 4 h in an incubator under sterile conditions at 37 °C in an atmosphere of 5% CO_2_. After incubation, the microplates were centrifuged at 1500× *g* for 10 min at room temperature. Then, the supernatant was carefully withdrawn, with the pipette tip not touching the bottom of the well, 0.15 mL of DMSO was added to each well, and the microplates were incubated for 10 min at 37 °C in an atmosphere of 5% CO_2_. The microplates were then incubated on a microplate shaker for 20 min with stirring at 200 rpm until the formazan crystals were completely dissolved. The optical density was estimated in each well at the formazan absorbance peak wavelength of 540 nm using a Spark^TM^ 10M multimode microplate reader (Tecan, Männedorf, Switzerland) according to the manufacturer’s protocol.

The cell survival rate was calculated by following the equation:

(1) $$Cell\;viability=\frac{A_{i}}{A_{0}}\times 100\%$$

where *A_i_* is the average optical density in the wells containing cells and the sample suspension; *A*_0_ is the average optical density in the control wells containing only cells, with the optical densities in the control wells containing the complete medium and the blank ones taken into account. 

#### 2.2.5. Inhibitory Dose Estimation and Statistical Analysis of Data

The Origin Pro version 8.5.0 SR1, Data Analysis, and Graphing software (OriginLab Corporation, Northampton, MA, USA, 2010) were used for the estimation of the inhibitory concentration and statistical analyses of the data (Student’s *t*-test). The results are presented as the mean and standard deviation for three independent experiments if not indicated otherwise.

## 3. Results and Discussion

### 3.1. Preparation and Characterization of Microstructures of Different Types

**Figure 1:**
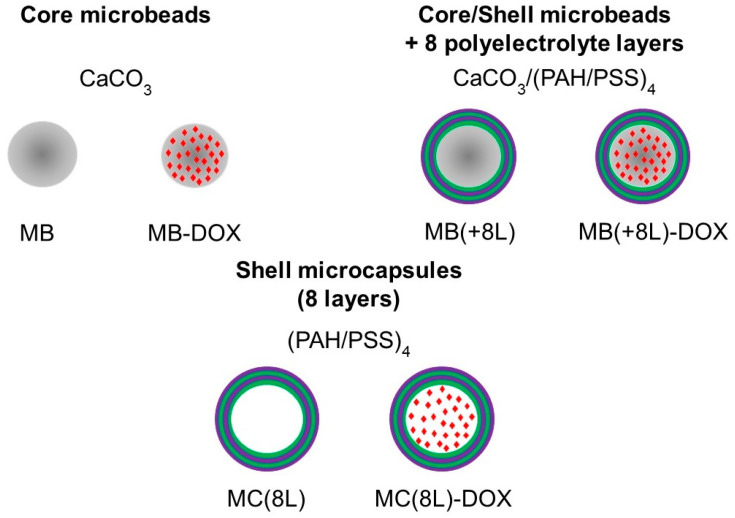
Synthesized microstructures. Designations: MB, core microbeads; MB-DOX, doxorubicin-containing core MBs with a regular spherical shape; MB(+8L), core/shell MBs with a shell of eight polyelectrolyte layers; MB(+8L)-DOX, doxorubicin-containing MB(+8L); MC(8L), soft hollow microcapsules with a shell of eight polyelectrolyte layers; and MC(8L)-DOX, MC(8L) loaded with doxorubicin.

In order to use the microstructures for targeted drug delivery, their size should be no more than several micrometers, and they should have well-defined shape and surface properties, ensuring optimal distribution, release kinetics, degradation rate, and elimination time [32,33]. In addition, the microstructure material should allow their loading with drug substances. Here, we engineered DOX-containing core microbeads with a regular spherical shape (MB-DOX), core/polymer-shell structures (MB(+8L)-DOX), and soft shell-type hollow microcapsules (MC(8L)-DOX). In addition, similar microstructures not containing DOX were synthesized and used as controls (Figure 1).

The size of the obtained microstructures was determined by the size of the synthesized calcium carbonate matrix core, which had good biocompatibility, biodegradability, and pyrogenicity. 

The core MBs represented calcium carbonate microparticles obtained by crystallization from mixed sodium carbonate and calcium chloride solutions. Glycerol was added to the reaction mixture as a thickener [21,22,34]. This approach yielded spherical microparticles (of the vaterite type) that were smaller than those synthesized without a thickener. The MBs obtained in this study had a porous structure, a narrow size distribution (~2.4 ± 0.5 μm), and a negative surface charge (−16.3 ± 0.8 mV); they were used as a matrix for obtaining highly homogeneous MB(+8L).

The subsequent layer-by-layer adsorption of oppositely charged polyelectrolytes onto the core yielded microparticles with several protective layers of polymers on the surface, as well as, after an additional procedure of core removal, hollow MC. The core/shell microstructures MB(+8L) were formed via layer-by-layer adsorption of oppositely charged polyelectrolytes, PAH and PSS, onto calcium carbonate MBs. This technique allowed for obtaining microstructures of uniform size, which is important in terms of their passive transport because carriers of the same size are transported and accumulated in the body uniformly. The size of the synthesized MB(+8L) was 2.5 ± 0.3 μm, and the surface charge was more negative (−32.1 ± 2.2 mV). Soft hollow microstructures (MC(8L)) were obtained by treating MB(+8L) with 0.5 M EDTA to dissolve the calcium carbonate core while preserving the polymer shell; the size and surface properties remained unchanged. However, the obtained MC(8L) lost the regular spherical structure, although they remained rounded.

The main advantage of the obtained microstructures is the possibility of controlled modification of the release of the loaded compounds, as well as the protection of these compounds from external factors that can cause their degradation.

The amphiphilicity of the anticancer drug DOX and the hydrophilicity of its salt form, DOX hydrochloride, preclude using standard approaches for its loading into the microcarriers. Currently, the most common approaches are the spontaneous loading of DOX [35] and its encapsulation at the stage of synthesis of these microcarriers, e.g., by the coprecipitation method [36]. It should be noted that DOX-loading methods that use only the aqueous phase are of particular interest because they do not require organic solvents, an oil phase, or special equipment for dispersion and emulsification. 

We used different microstructures, for which the optimal methods of DOX loading were also different. Specifically, the coprecipitation method was optimal for loading DOX into MB and MB(+8L), whereas the spontaneous loading ensured the highest loading efficiency in the case of MC(8L). Loading the same quantities of DOX into all microcarriers was also important for our subsequent experiments on cell viability using the same number of microstructures per cell with a normalized DOX concentration. 

**Figure 2:**
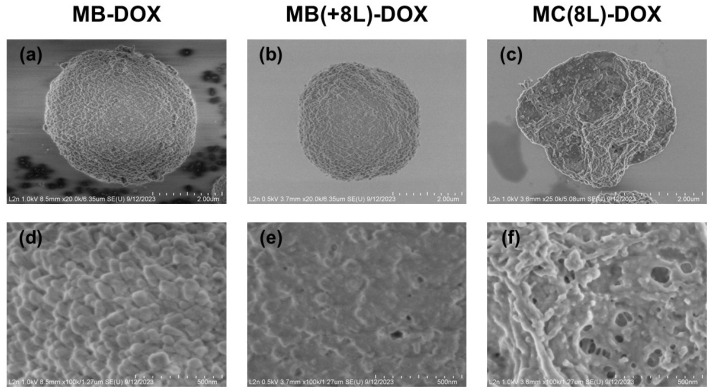
Scanning electron microscopy images of the microstructures loaded with doxorubicin. (**a**,**d**) Core microbeads (MB-DOX); (**b**,**e**) microbeads coated with eight polyelectrolyte layers (MB(+8L)-DOX); (**c**,**f**) microcapsules with a shell of eight polyelectrolyte layers (MC(8L)-DOX).

The synthesized MB-DOX had a porous structure (Figure 2a,d), a narrow size distribution (2.7 ± 0.5 μm), and a negative ζ-potential (−11.3 ± 1.8 mV). The efficiency of DOX loading by this method was 76.4 ± 2.9% (Table 1). 

The MB-DOX was used as a matrix for the engineering of MB(+8L)-DOX. The resultant MB(+8L)-DOX (Figure 2b,e) were within the same size range as the original MBs (*p* > 0.05, Student’s *t*-test), 2.7 ± 0.3 μm. In order to obtain MB(+8L)-DOX with a standardized amount of DOX per microcarrier, it was necessary to take into account the loss of DOX during the application of the polyelectrolyte shell. However, an experimental estimation showed that the loss of DOX was negligible (2–6%). The efficiency of DOX loading by this method was 74.3 ± 4.8 (Table 1). 

The preliminarily fabricated control MC(8L) (2.7 ± 0.4 μm) was used for obtaining MC(8L)-DOX by spontaneous loading. The mean size of the MC(8L)-DOX (Figure 2c,f) did not differ significantly from that of the original MCs (2.7 ± 0.4 μm) (*p* > 0.05, Student’s *t*-test). The efficiency of DOX encapsulation by this method was 73.9 ± 3.9% (Table 1).

Thus, the coprecipitation and spontaneous loading methods used in this study for preparing DOX-containing microstructures provided a higher efficiency of DOX loading into microstructures in the aqueous phase compared to the previously reported ones (~29–44% [36,37] and ~50% [20], respectively).

### 3.2. Release of Doxorubicin from the Microcarriers

**Figure 3:**
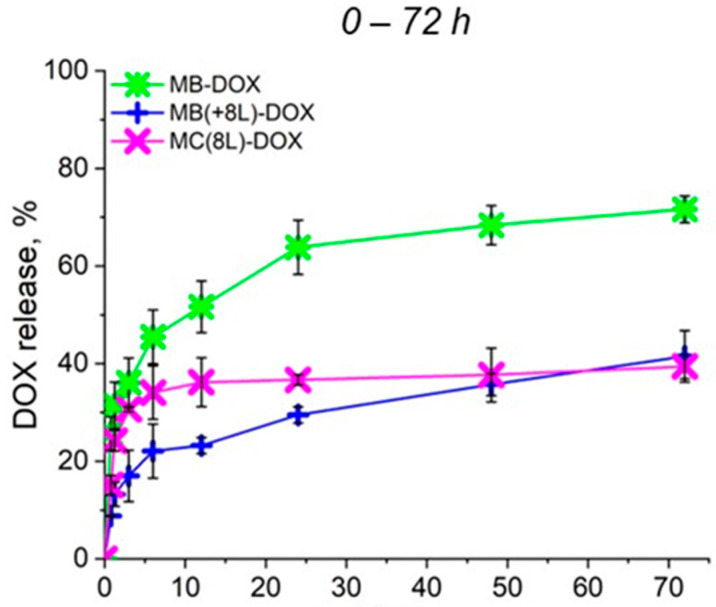
Profiles of doxorubicin release from microcarriers at pH 7.4 during 72 h. Designations: MB-DOX, core microbeads containing doxorubicin; MB(+8L)-DOX, core microbeads containing doxorubicin and coated with eight polyelectrolyte layers; MC(8L)-DOX, microcapsules with a shell of eight polyelectrolyte layers containing doxorubicin.

To further analyze the synergistic effect of the microcarrier structure and released DOX on cell viability, the rate of DOX release from the prepared microcarriers under the conditions used for the cell culture at 37 °C and a pH of 7.4 was preliminarily evaluated (Figure 3). As seen in Figure 3, a prolonged release of DOX from all microcarriers was demonstrated. In the case of MBs, an explosive release was observed, but the cumulative release of DOX did not exceed 75% within 72 h, as we have already shown earlier [22]. The plots shown in Figure 3 demonstrate that the polyelectrolyte shell inhibited the explosive release of the drug from MB(+8L) and MC(8L) at the initial stages. The cumulative release of DOX from MB(+8L) and MC(8L) did not exceed 40% within 72 h. Slow release of the anticancer compound at the physiologic pH may facilitate the preservation of the functional properties of the compound, as well as reduce toxicity to healthy cells of the human body. Apparently, the core/shell microstructures and MCs are the most promising drug carriers because they exhibited a longer release of DOX compared to the MBs.

### 3.3. Cell Viability in the Presence of the Microstructures

The main objective in the preparation of microcarriers for antitumor therapy is to reduce the toxic effect on healthy cells while preserving or enhancing the toxic effect on tumor cells. Thus, cell viability analysis is essential to assess the applicability of microcarriers for in vitro drug delivery, as well as to evaluate the functional activity of the compound loaded into the microcarriers. We analyzed the cytotoxicity of DOX-containing microstructures in comparison with the cytotoxicity of DOX-free microstructures by the MTT method using tumor cells (epidermoid carcinoma A-431 cells) and immune cells (THP-1 human peripheral blood monocytes differentiated into macrophages).

The viability of cells in the presence of different microcarriers was assessed under the same conditions by varying the microcarrier-to-cell ratio from 1:1 to 50:1. The loading conditions for different types of microcarriers were preliminarily determined in order to load the same amounts of DOX into different types of microcarriers (Table 2).

**Figure 4:**
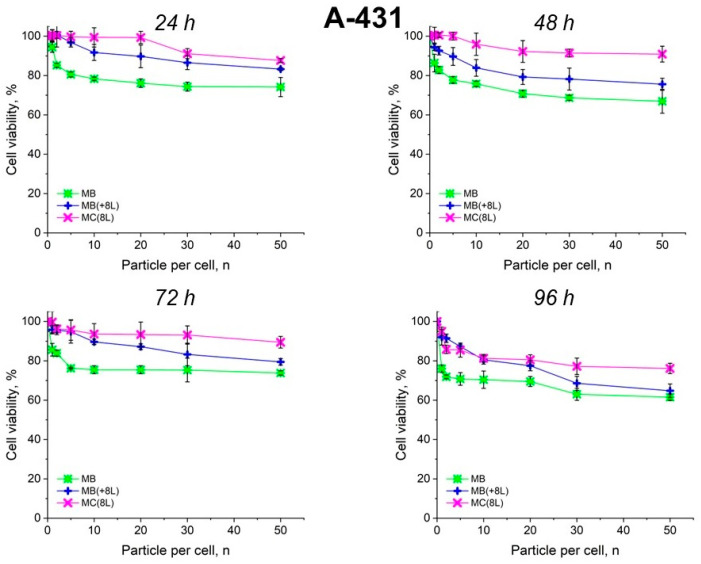
Viability of A-431 cells as estimated by the MTT assay. Designations: MB, core microbeads; MB(+8L), core microbeads coated with eight polyelectrolyte layers; MC(8L), microcapsules with a shell of eight polyelectrolyte layers.

**Figure 5:**
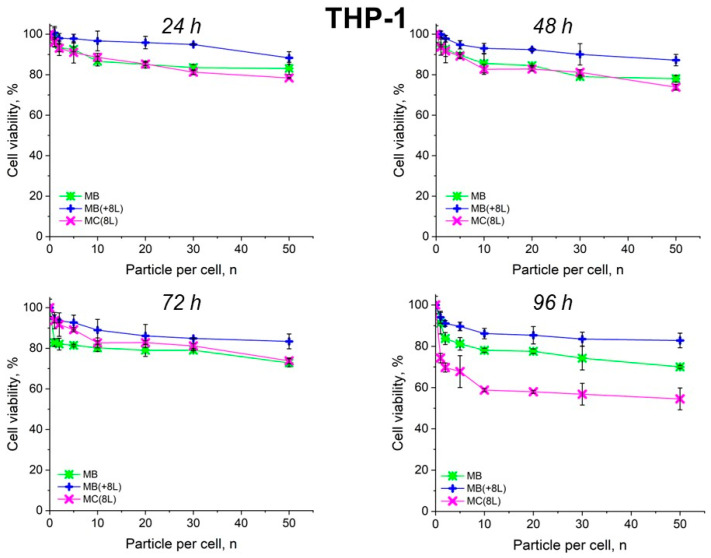
Viability of THP-1 cells as estimated by the MTT assay. Designations: MB, core microbeads; MB(+8L), core microbeads coated with eight polyelectrolyte layers; MC(8L), microcapsules with a shell of eight polyelectrolyte layers.

The results showed that the control DOX-free microstructures insignificantly affected the proliferation rate of both the tumor and immune cells. A slight decrease in cell viability after prolonged incubation to 70–80%, depending on the type of microcarriers, was observed. It is also of interest that spherical microparticles with a regular structure (MBs) had the highest cytotoxic effect on tumor cells (*p* < 0.05, Student’s *t*-test), while the maximum cytotoxic effect on immune cells was exerted by soft hollow MCs (*p* < 0.05, Student’s *t*-test), whose wall consisted of eight polyelectrolyte layers. At the same time, spherical microparticles with a regular structure of the core that was coated with eight polyelectrolyte layers (MB(+8L)) were practically nontoxic for immune cells (*p* < 0.05, Student’s *t*-test) (Figure 4 and Figure 5; Table 3 and Table 4).

**Figure 6:**
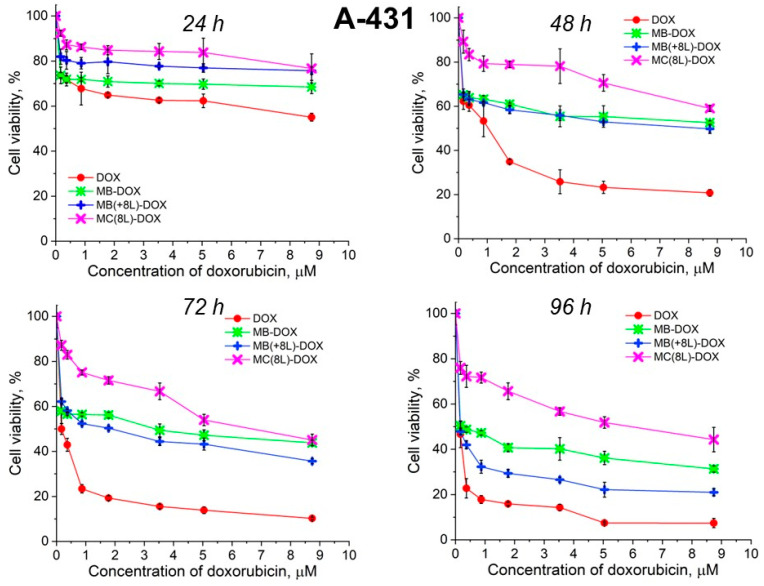
Viability of A-431 cells as estimated by the MTT assay. Designations: DOX, doxorubicin; MB-DOX, core microbeads containing doxorubicin; MB(+8L)-DOX, core microbeads containing doxorubicin and coated with eight polyelectrolyte layers; MC(8L)-DOX, microcapsules with a shell of eight polyelectrolyte layers containing doxorubicin.

**Figure 7:**
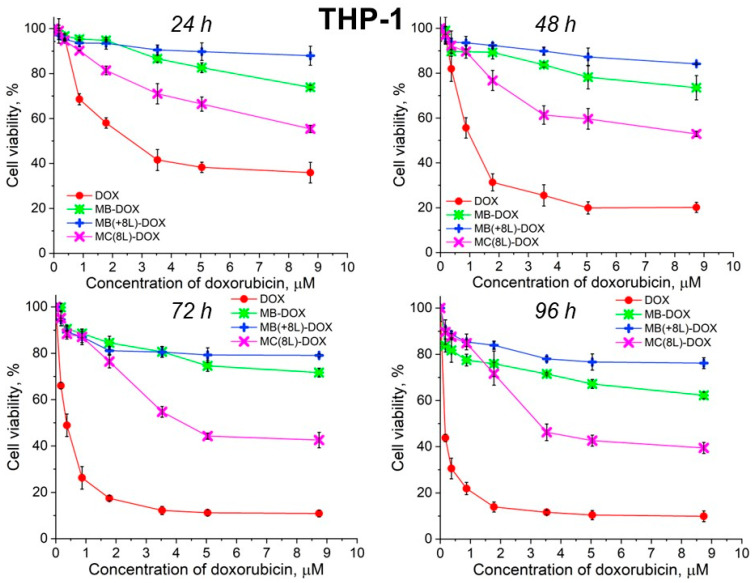
Viability of THP-1 cells as estimated by the MTT assay. Designations: DOX, doxorubicin; MB-DOX, core microbeads containing doxorubicin; MB(+8L)-DOX, core microbeads containing doxorubicin and coated with eight polyelectrolyte layers; MC(8L)-DOX, microcapsules with a shell of eight polyelectrolyte layers containing doxorubicin.

Unencapsulated DOX was highly toxic for both A-431 and THP-1 cells, with the survival rate of the macrophages in the presence of free DOX being lower than that of the tumor cells (Figure 6 and Figure 7, Table 5 and Table 6).

It was found that the encapsulation of DOX in microcarriers considerably increased the survival rate of both the tumor and immune cells. At the same time, the toxic effect of encapsulated DOX on the cancer cells was delayed, but it was stronger than that on immune cells. This can be explained by its more rapid transport into cancer cells and the lack of attenuation of the toxic effect of the transported DOX by the drug resistance mechanisms of cancer cells. The differences between the cancer cell cytotoxicities of free DOX and DOX encapsulated in different microcarriers increased with time, which was due to the difference between the rates of DOX release from different types of microcarriers (Figure 6 and Figure 7, Table 5 and Table 6). On the other hand, the delayed toxic effect of encapsulated DOX on tumor cells was comparable to the effect of unencapsulated DOX (*p* > 0.05, Student’s *t*-test) (Figure 6, Table 5).

An interesting finding was that the microcarriers themselves influenced the cytotoxic effect of DOX. MC(8L)-DOX were less toxic for tumor cells compared to MB-DOX and MB(+8L)-DOX (*p* < 0.05, Student’s *t*-test) (Figure 6, Table 5). The cytotoxic effect of MB(+8L)-DOX during the first 24 h was slightly weaker than that of MC(8L)-DOX. However, the cytotoxicity of MB(+8L)-DOX was similar to that of MB-DOX after 48 h of incubation (*p* > 0.05, Student’s *t*-test) and became stronger than the cytotoxicities of all other microstructures after 96 h of incubation (*p* < 0.05, Student’s *t*-test). This was probably because their core/polymer-shell structure favored the biphasic release of encapsulated DOX and was more rigid compared to MC(8L)-DOX [28]. 

In contrast, MB-DOX and MB(+8L)-DOX exhibited lower cytotoxicity towards the macrophages than MC(8L)-DOX did (*p* < 0.05, Student’s *t*-test) (Figure 7, Table 6), even upon prolonged incubation. This can be explained by the soft structure of MC(8L)-DOX, which determined their more rapid uptake by macrophages compared to cancer cells [35], probably because they more readily change shape when uptaken by the cells [28]. It is also possible that macrophages and cancer cells use different mechanisms for uptaking different types of microstructures: micropinocytosis or clathrin- or caveolin-mediated phagocytosis [38]. 

Thus, the study of the viability of A-431 tumor cells and differentiated THP-1 human macrophages in the presence of the microstructures loaded with DOX has shown that encapsulation of this antitumor drug decreases its cytotoxicity against normal cells and delays its toxic effect against tumor cells. The DOX-containing microstructures can provide a longer action of DOX on tumor cells, comparable in strength to that of unencapsulated DOX, thus reducing its nonselective side effects on the body while preserving its pharmacological activity. The rigid microstructures with a polymer shell (MB(+8L)-DOX) are the most attractive among the microstructures studied because they exhibit lower cytotoxicity against normal human cells, even upon prolonged incubation, and a strong delayed cytotoxic effect against tumor cells. 

The results of this study could serve as a basis for the development of new drug delivery systems because the approach used here allows for obtaining microstructures with different physical and chemical properties. The optimal size of the microstructures for intravenous/intramuscular administration is known to be about several micrometers, their optimal shape being spherical [39,40]. The size can also determine the biodistribution of microstructures in different organs (spleen, liver, or lungs) after their injection [39,41]. Furthermore, the presence of a polyelectrolyte shell is expected to be important because it can significantly increase the circulation time of the microstructures in the bloodstream and provide a controlled prolonged release of the loaded drug from the microstructures in the vicinity of cancer cells. All these properties together could play a key role in future in vivo applications. Therefore, the next stage of our study will be aimed at evaluating the in vivo behavior of the microstructures and determining the parameters that affect the efficacy of the microstructures as drug delivery agents for the treatment of cancer.

## 4. Conclusions

The results of this study show that the microcarrier structural characteristics, such as the stiffness and regularity of the microcarrier structure, should be taken into account in the development of delivery systems for antitumor drugs. It has been demonstrated that regular rigid spherical microcarriers containing an additional protective shell of oppositely charged polyelectrolyte layers on the surface are promising drug delivery tools that can be adapted for use as antitumor therapeutic agents. Conversely, softer hollow microcapsules of the same size are highly cytotoxic for human macrophages and may induce undesirable effects on the immune system. The core/shell microstructures with an eight-layer polyelectrolyte shell designed in this study represent a promising platform for further development of theranostic agents for the diagnosis and treatment of tumors.

## Figures and Tables

**Table 1:** Efficiency of doxorubicin loading into the engineered microcarriers.

Sample ^1^	Loading Efficiency, %	Amount of DOX per Microcarrier, µg
MB-DOX	76.4 ± 2.9	2 × 10^−6^ ± 5.8 × 10^−7^
MB(+8L)-DOX	74.3 ± 4.8	1.96 × 10^−6^ ± 1.3 × 10^−7^
MC(8L)-DOX	73.9 ± 3.9	1.9 × 10^−6^ ± 1 × 10^−7^

^1^ Microstructures loaded with doxorubicin (DOX): MB-DOX, microbeads; MB(+8L)-DOX, microbeads coated with eight polyelectrolyte layers; MC(8L)-DOX, microcapsules with eight polyelectrolyte layers shell.

**Table 2:** Doxorubicin concentrations corresponding to different numbers of microcarriers.

Number of Particles per Cell	1	2	5	10	20	30	50
Average Concentration of DOX ^1^, µM	0.175 ± 0.004	0.371 ± 0.008	0.878 ± 0.019	1.79 ± 0.039	3.55 ± 0.075	5.06 ± 0.111	8.78 ± 0.192

^1^ Doxorubicin (DOX).

**Table 3:** Inhibitory concentrations of microcarriers for A-431 cells.

Sample ^1^	IC Values, Particles per Cell			
Agent Type	24 h	48 h	72 h	96 h
MB	IC_20_ = 5.5 ± 0.03	IC_20_ = 3.5 ± 0.06	IC_20_ = 3.25 ± 0.05	IC_20_ = 0.5 ± 0.2
MB(+8L)	IC_20_ –	IC_20_ = 33.3 ± 0.04	IC_20_ = 10.8 ± 0.08	IC_20_ = 11.01 ± 0.03
MC(8L)	IC_20_ –	IC_20_ –	IC_20_ –	IC_20_ = 22.2 ± 0.05

^1^ MB, core microbeads; MB(+8L), core microbeads coated with eight polyelectrolyte layers; MC(8L), microcapsules with a shell of eight polyelectrolyte layers.

**Table 4:** Inhibitory concentrations of microcarriers for THP-1 cells.

Sample ^1^	IC Values, Particles per Cell			
Agent Type	24 h	48 h	72 h	96 h
MB	IC_20_ –	IC_20_ = 42.4 ± 0.08	IC_20_ = 22.5 ± 0.06	IC_20_ = 7.2 ± 0.02
MB(+8L)	IC_20_ –	IC_20_ –	IC_20_ –	IC_20_ –
MC(8L)	IC_20_ = 32.3 ± 0.03	IC_20_ = 31.2 ± 0.05	IC_20_ = 6.2 ± 0.09	IC_20_ = 0.6 ± 0.03

^1^ MB, core microbeads; MB(+8L), core microbeads coated with eight polyelectrolyte layers; MC(8L), microcapsules with a shell of eight polyelectrolyte layers.

**Table 5:** Inhibitory concentrations of doxorubicin for A-431 cells.

Sample ^1^	IC Values			
Agent Type	24 h	48 h	72 h	96 h
DOX	IC_20_ = 0.06 ± 0.017 IC_50_ –	IC_20_ = 0.03 ± 0.02 IC_50_ = 1.09 ± 0.07	IC_20_ = 0.018 ± 0.04 IC_50_ = 0.17 ± 0.03	IC_20_ = 0.01 ± 0.05 IC_50_ = 0.085 ± 0.04
MB-DOX	IC_20_ = 0.08 ± 0.03 IC_50_ –	IC_20_ = 0.04 ± 0.02 IC_50_ –	IC_20_ = 0.02 ± 0.04 IC_50_ = 3.22 ± 0.03	IC_20_ = 0.005 ± 0.002 IC_50_ = 0.15 ± 0.015
MB(+8L)-DOX	IC_20_ = 0.36 ± 0.06 IC_50_ –	IC_20_ = 0.08 ± 0.05 IC_50_ = 7.56 ± 0.43	IC_20_ = 0.04 ± 0.025 IC_50_ = 1.71 ± 0.02	IC_20_ = 0.02 ± 0.003 IC_50_ = 0.12 ± 0.07
MC(8L)-DOX	IC_20_ = 5.14 ± 0.04 IC_50_ –	IC_20_ = 0.75 ± 0.35 IC_50_ –	IC_20_ = 0.47 ± 0.08 IC_50_ = 6.24 ± 0.52	IC_20_ = 0.08 ± 0.04 IC_50_ = 5.33 ± 0.07

^1^ DOX, doxorubicin; MB-DOX, microbeads containing doxorubicin; MB(+8L)-DOX, microbeads containing doxorubicin and coated with eight polyelectrolyte layers; MC(8L)-DOX, microcapsules with a shell of eight polyelectrolyte layers containing doxorubicin.

**Table 6:** Inhibitory concentrations of doxorubicin for THP-1 cells.

Sample ^1^	IC Values			
Agent Type	24 h	48 h	72 h	96 h
DOX	IC_20_ = 0.54 ± 0.05 IC_50_ = 2.83 ± 0.06	IC_20_ = 0.38 ± 0.08 IC_50_ = 0.91 ± 0.04	IC_20_ = 0.19 ± 0.06 IC_50_ = 0.35 ± 0.05	IC_20_ – IC_50_ = 0.17 ± 0.04
MB-DOX	IC_20_ = 4.89 ± 0.03 IC_50_ –	IC_20_ = 4.81 ± 0.03 IC_50_ –	IC_20_ = 1.78 ± 0.1 IC_50_ –	IC_20_ = 0.81 ± 0.06 IC_50_ –
MB(+8L)-DOX	IC_20_ – IC_50_ –	IC_20_ – IC_50_ –	IC_20_ = 3.45 ± 0.04 IC_50_ –	IC_20_ = 3.44 ± 0.34 IC_50_ –
MC(8L)-DOX	IC_20_ = 1.54 ± 0.2 IC_50_ –	IC_20_ = 1.61 ± 0.03 IC_50_ –	IC_20_ = 1.25 ± 0.04 IC_50_ = 4.05 ± 0.01	IC_20_ = 0.94 ± 0.02 IC_50_ = 2.55 ± 0.6

^1^ DOX, doxorubicin, MB-DOX, core microbeads containing doxorubicin; MB(+8L)-DOX, core microbeads containing doxorubicin and coated with eight polyelectrolyte layers; MC(8L)-DOX, microcapsules with a shell of eight polyelectrolyte layers containing doxorubicin.

## Data Availability

The data presented in this study are available on request from the corresponding authors.
